# Determination of Crizotinib in Mouse Tissues by LC-MS/MS and Its Application to a Tissue Distribution Study

**DOI:** 10.1155/2020/8837254

**Published:** 2020-12-18

**Authors:** Fang Zhao, Yuan Wei, Yiming Yan, Han Liu, Sitong Zhou, Bo Ren, Ruijuan Liu

**Affiliations:** ^1^Department of Pharmacy, The First Affiliated Hospital of Zhengzhou University, Zhengzhou 450052, China; ^2^Henan Key Laboratory of Precision Clinical Pharmacy, Zhengzhou 450052, China

## Abstract

Toxicity induced by crizotinib, a small-molecule tyrosine kinase inhibitor, is a significant clinical issue during treatment. A tissue distribution study is required to explore the organs affected by this molecule. In this study, a simple liquid chromatography tandem mass spectrometry method was developed and validated for the determination of crizotinib in various mouse tissues. Mouse tissue homogenates were processed by protein precipitation with methanol, and apatinib was chosen as the internal standard. The analytes were separated on a Phenomenex Kinetex C_18_ (50 mm × 2.1 mm, 2.6 *μ*m) column with gradient elution using methanol and 0.3% formic acid water solution. Tandem mass spectrometric detection was conducted using multiple reaction monitoring via an electrospray ionization source in the positive mode. The monitored ion transitions were *m*/*z* 450.1 ⟶ 260.2 for crizotinib and *m*/*z* 398.2 ⟶ 212.0 for apatinib. The problem of the severe carryover effect was successfully resolved. The method was validated and applied to a tissue distribution study of crizotinib in mice, which was reported for the first time. The results of the study showed that the main target organs of crizotinib were the lung, liver, and spleen, and a high concentration of crizotinib was found in the gastrointestinal tract. This study offers a reliable method for quantifying crizotinib and provides a basis for further research on crizotinib toxicity.

## 1. Introduction

**Figure 1:**
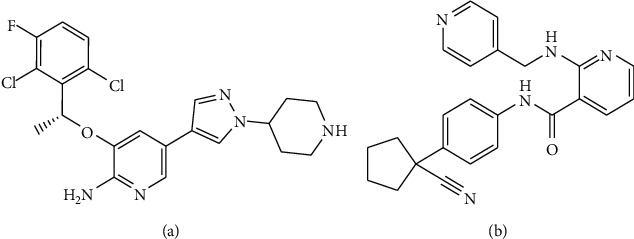
The chemical structures of (a) crizotinib and (b) apatinib (IS).

Crizotinib (Figure 1(a)) is an oral multitargeted receptor tyrosine kinase inhibitor that targets anaplastic lymphoma kinase (ALK), ROS proto-oncogene 1 (ROS1), and hepatocyte growth factor MET receptor tyrosine kinases [1, 2]. Crizotinib was approved by the FDA as a first-line treatment of ALK-rearranged nonsmall cell lung cancer (NSCLC) in 2011 [1] and was approved in the United States and European Union for the therapy of patients with ROS1-positive advanced NSCLC in 2016 [3]. To date, crizotinib remains the only approved, standard first-line therapy for ROS1-rearranged NSCLC [4, 5] and is highly effective in the clinic [6]. Furthermore, it represents a clinically meaningful treatment option for NSCLC with MET  alterations [2]. Compared with chemotherapy, crizotinib showed a better therapeutic effect in ALK-positive lung cancer [7]. Therefore, crizotinib is used globally for NSCLC treatment. However, the toxicity induced by crizotinib is a challenging clinical problem during the treatment of NSCLC [8–10], although crizotinib is safer and more effective than chemotherapy. To explore the mechanism of its toxicity and preventive measures, it is necessary to investigate the tissue distribution of crizotinib at toxic doses.

An accurate and reliable bioanalytical method is needed to detect crizotinib in different biosamples for tissue distribution. To our knowledge, several liquid chromatography tandem mass spectrometry (LC-MS/MS) methods have been developed for the determination of crizotinib in human plasma for therapeutic drug monitoring [11–14] or pharmacokinetic studies [15]. In addition, Qiu et al. [16] and Sparidans et al. [17] reported two LC-MS/MS methods for the quantification of crizotinib in rat or mouse plasma and applied it to pharmacokinetic studies. However, no successful method for the quantitative determination of crizotinib in mouse tissues has been reported thus far. Moreover, there have been no reports on the tissue distribution of crizotinib at toxic doses in mice.

In this study, a simple liquid chromatography tandem mass spectrometry method was developed and validated for the determination of crizotinib in various mouse tissues. The method was successfully applied to the tissue distribution of crizotinib in mice after the oral administration of crizotinib.

## 2. Materials and Methods

### 2.1. Chemicals and Reagents

Crizotinib (purity >99%) for the analytical method and dosage administration was purchased from Dalian Meilun Biology Technology Co., Ltd. (Dalian, China). The reference standards of apatinib (Figure 1(b), internal standard (IS) purity ≥99%) and formic acid (chromatographic grade) were purchased from Aladdin Industrial Corporation (Shanghai, China). Sodium carboxymethylcellulose (CMC-Na) was purchased from Sinopharm Chemical Reagent Co., Ltd. (Shanghai, China). Methanol, acetonitrile, and isopropanol were all HPLC grade and were purchased from Fisher Scientific (Shanghai, China). Deionized water was purified using a Milli-Q system (Millipore, Milford, MA, USA).

### 2.2. Animals

Institute of Cancer Research mice (20 ± 2 g, half male and half female) were obtained from Beijing Vital River Laboratory Animal Technology Co., Ltd. (Beijing, China). They were kept in an environmentally controlled breeding room for one week before starting the experiments and were fed standard laboratory food and water. The mice were fasted with free access to water for at least 10 h before the administration of the drugs. Animal studies were performed in accordance with the Guide for the Care National Institutes of Health. Ethical approval for the animal experiment was obtained from the Animal Ethics Committee of the First Affiliated Hospital of Zhengzhou University (Zhengzhou, China).

### 2.3. Instrumentation and Conditions

The LC-MS/MS system consisted of an ExionLC™ analytical ultrahigh-performance liquid chromatography (UPLC) system (AB Sciex, USA) and a Qtrap 4500 triple quadrupole mass spectrometer (AB Sciex, USA) equipped with Turbo ion spray interface operation in the electrospray ionization (ESI) mode. Chromatographic separation was performed on a Kinetex C_18_ column (2.6 *μ*m, 50 mm × 2.1 mm, i.d., Phenomenex, USA) at 40°C with a mobile phase of methanol (solvent A) and 0.3% formic acid water solution (solvent B), and the flow rate was 0.3 mL/min. The linear gradient program was as follows: 0 to 0.2 min, 10% A; 0.2 to 0.5 min, 10 to 60% A; 0.5 to 1.9 min, 60% A; 1.9 to 2.1 min, 60 to 100% A; 2.1 to 3.1 min, 100% A; 3.1 to 3.5 min, 100 to 10% A; and 3.5 to 5.0 min, 10% A. The volume of injection was 1 *μ*L, and the autosampler temperature was maintained at 4°C. A methanol/acetonitrile/isopropanol (1 : 1 : 1, v/v/v) solution containing 5% formic acid was selected as the needle rinse solution.

The mass spectrometer detector was set as multiple reaction monitoring (MRM) in the positive ion mode. The MRM fragmentation transitions were *m*/*z* 450.1 ⟶ *m*/*z* 260.1 for crizotinib and *m*/*z* 398.2 ⟶ *m*/*z* 212.0 for IS. The dwell time for each transition was 100 ms. The instrument was operated with the ion spray voltage set at 5.0 kV and the heater gas temperature set to 500°C. The nebulizer gas (gas (1)) and heater gas (gas (2)) were both maintained at 50 psi. In addition, a curtain gas of 30 psi and a collision gas of medium were used. The mass parameters for both crizotinib and IS were optimized as follows: entrance potential (EP) was 10 V; collision cell exit potential (CXP) was 6.5 V; and collision energy (CE) was 34 V. The declustering potential (DP) for crizotinib and IS was set to 110.5 V and 118 V, respectively. Data acquisition was conducted using Analyst 1.6.2 software (Applied Biosystems, USA).

### 2.4. Preparation of Calibration Standards and Quality Control Samples

The stock solutions of crizotinib and IS were prepared by dissolving accurately weighed amounts of the reference substances in methanol to yield concentrations of 1.0 mg/mL. Working solutions of crizotinib at the desired concentration for the preparation of calibration standards and quality control (QC) samples were obtained by serially diluting stock solutions using methanol. The 400 ng/mL working solution was prepared in methanol. All solutions were stored at −20°C until use.

Calibration standards and QC samples were prepared by spiking the appropriate volumes of the working solutions with blank (drug-free) mouse tissue homogenates. The blank tissue homogenates were prepared with the liver, lung, kidney, spleen, fat, stomach, large intestine, small intestine, heart, muscle, and brain, respectively. The preparation method was the same as the method adopted in Section 2.7. The final concentration levels of the standard curve samples were 20–8000 ng/mL. The QC samples were prepared at concentrations of 60, 600, and 6400 ng/mL for crizotinib.

### 2.5. Sample Preparation

After adding 50 *μ*L aliquots of mouse tissue homogenate to a 1.5 mL centrifuge tube, 5 *μ*L of IS solution (400 ng/mL) was added, and the mixture was vortexed for 10 s. Then, 200 *μ*L methanol was added to the mixture for protein precipitation and extraction of the analytes, vortex-mixed for 3 min, and centrifuged at 4°C at 14000 rpm for 10 min. The supernatant was separated and mixed with an equal volume of 60% (v/v) methanol-water solution. Finally, the mixture was transferred into an autosampler vial, and 1 *μ*L was injected for UPLC-MS/MS analysis.

### 2.6. Method Validation

Method validations were performed using the UPLC-MS/MS method according to the guidelines set by the United States Food and Drug Administration (FDA) [18] and the Chinese Pharmacopoeia Commission [19] for bioanalytical method validation. The partial method validation could be performed when changes in the matrix within species were occurred in bioanalytical method [18]. In this study, the full-method validations, including selectivity, linearity, precision and accuracy, matrix effect, recovery, carryover effect, stability, and dilution integrity, were performed on liver and small intestine samples. Partial method validations were conducted for the other tissue samples.

### 2.7. Tissue Distribution Study

Thirty-six mice were randomly divided into six groups (six mice per group, half male and half female). After fasting overnight, the mice were administered a single dose of 500 mg/kg crizotinib through oral gavage. The crizotinib was prepared in 0.5% carboxymethyl cellulose sodium (CMC-Na) to make a suspension of 50 mg/mL. The mice were euthanized by decapitation, and different tissues were harvested at each time point. Tissues including the brain, heart, liver, lung, kidney, spleen, fat, muscle, stomach, large intestine, and small intestine were dissected, washed with saline, and blotted with filter paper. Tissue samples were homogenized in ice-cold saline (1 : 50, w/v, g/mL for the liver, lung, kidney, spleen, fat, stomach, large intestine, and small intestine; 1 : 30, w/v for the heart and muscle; and 1 : 10, w/v for the brain). These tissue homogenates were stored at −80°C, allowed to thaw at room temperature, and vortex-mixed before processing.

In this study, the animal equivalent dose was calculated according to the guidelines set by the US FDA [20]. The equivalent dose of crizotinib in mice was 102.5 mg/kg, and it was obtained by multiplying the dose in humans (500 mg/day, assumed 60 kg human) by the body surface area conversion factor (12.3). The toxic dosage (500 mg/kg) of crizotinib in mice was about five times that of the equivalent dose of crizotinib in mice. The toxic dosage has been investigated and verified in another study (data not shown).

## 3. Results and Discussion

### 3.1. Method Development and Optimization

#### 3.1.1. Optimization of Mass Spectrometry Conditions

**Figure 2:**
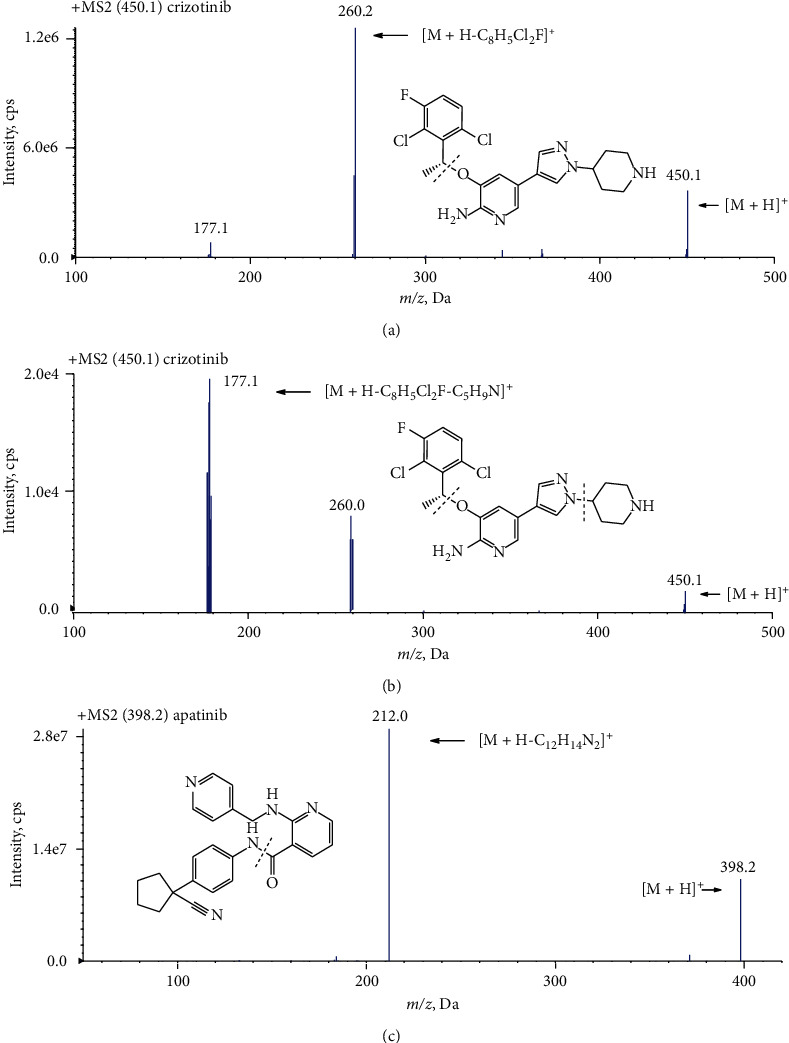
Product ion mass spectra of crizotinib (a, b) and apatinib (c) in positive mode and their proposed fragmentation patterns.

To optimize the mass spectrometric conditions, the standard solutions of crizotinib and IS were injected into the mass spectrometer. The positive-ion ESI mode was performed, considering that nitrogen is present in the chemical structures of crizotinib. In the Q1 full-scan mass spectra, the predominant ion of crizotinib was the protonated molecule ion [*M* + *H*]^+^ at *m*/*z* 450.1. The MRM mode was chosen because of its better specificity and sensitivity. Interestingly, the product ion mass spectrum of crizotinib showed that the main fragment ion was *m*/*z* 260.2 with low CE (approximately 34 eV, Figure 2(a)), whereas the main fragment ion was m/*z* 177.1 with high CE (about 45 eV, Figure 2(b)). The parameters of MS/MS were optimized, and the results showed that MRM transition of *m*/*z* 450.1 ⟶ *m*/z 260.2 offered a better mass response. The MRM reaction of the IS at m/*z* 398.2 ⟶ *m*/z 212.0 was used for quantification. The product ion mass spectra and the proposed fragmentation patterns of crizotinib and IS are shown in Figure 2.

#### 3.1.2. Optimization of Liquid Chromatographic Conditions

The chromatographic conditions, including the mobile phase composition and chromatographic column types, were optimized to achieve good chromatographic peak shapes, resolution, and proper retention time for the analytes according to the methods reported before [21–23]. After many tests, methanol with 0.3% formic acid using a gradient elution was chosen as the mobile phase. The gradient program was set as follows at first: 0 to 0.2 min, 10% A; 0.2 to 0.5 min, 10 to 60% A; 0.5 to 1.9 min, 60% A; 1.9 to 2.2 min, 60 to 10% A; and 2.2 to 4.0 min, 10% A. The column used for this method was an Acquity UPLC HSS T3 column (1.8 *μ*m, 100 mm × 2.1 mm i.d., Waters). Under that chromatographic conditions, crizotinib and IS exhibited good chromatographic behavior.

**Figure 3:**
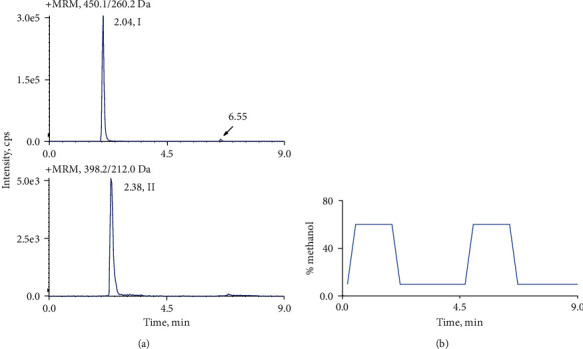
Chromatogram of the elution of a high-quality control sample using a “duplicated” solvent gradient: (a) crizotinib (I) and apatinib (II) and (b) the “duplicated” gradient.

Unfortunately, the carryover effect was detected for crizotinib under the initial chromatographic conditions. Carryover is a ubiquitous problem in the LC-MS bioanalytical method, and it limits accurate quantitation. Studies have demonstrated that carryover may be caused by the autosampler or LC column [24–26]. In this study, the source of carryover was examined using a “duplicated” solvent gradient method based on a previous study [26]. The “duplicated” solvent gradient includes two gradients for one analysis, which means that an injection is eluted by the first gradient, and the second gradient is performed without sample injection. The results (Figure 3) showed that the peak (6.55 min) obtained by the second gradient was lower than that (2.04 min) obtained by the first gradient, which indicated that the carryover was caused by both the autosampler and column. Therefore, possible solutions need to be tested to eliminate the carryover effect from the autosampler and column. We tried several needle rinse solvents (acetonitrile, methanol, and isopropanol) individually as well as with various combinations of these solvents. Finally, a methanol/acetonitrile/isopropanol (1 : 1 : 1, v/v/v) solution containing 5% formic acid was selected as the needle rinse solvent to clean the autosampler system. Furthermore, a wash step with a high organic phase ratio (100% methanol) added to the initial gradient program was performed to clean the column and reduce carryover from the column. The final gradient program was the one mentioned in Section 2.3. Although the carryover effect was improved in some way, it still could not meet the requirements of the bioanalytical method. Then, other kinds of columns, including Agela Venusil MP C_18_ column and Kinetex C_18_ column, were tested. Ultimately, a Kinetex C_18_ column (2.6 *μ*m, 50 mm × 2.1 mm, i.d., Phenomenex, USA) was chosen which provided the minimum carryover effect for crizotinib.

The differences between the current method and the plasma methods [11–17] reported mainly include sample preparation, mobile phase composition, gradient program, and linear range of crizotinib. The detailed differences are listed in Table 1.

### 3.2. Method Validation

#### 3.2.1. Selectivity

**Figure 4:**
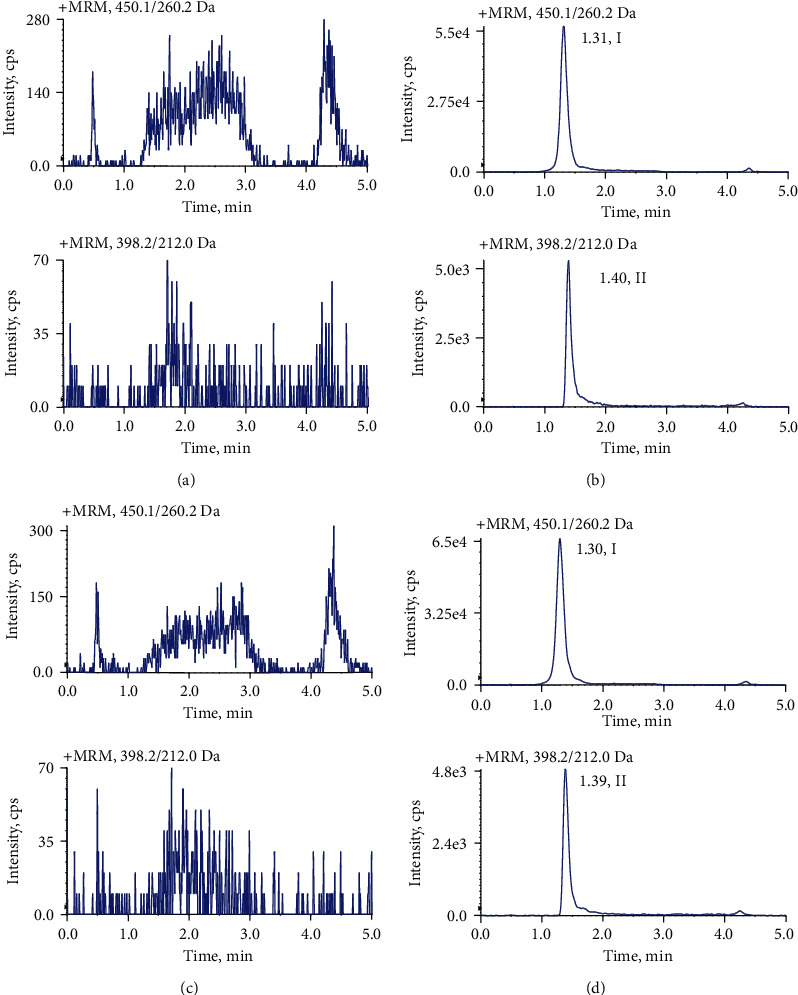
Typical MRM chromatograms of crizotinib (I) and apatinib (II, IS). (a) Blank liver homogenate sample; (b) liver tissue sample obtained from a mouse at 8 h after intragastric administration of 500 mg/kg crizotinib; (c) blank small intestine homogenate sample; and (d) small intestine tissue sample obtained from a mouse at 8 h after intragastric administration of 500 mg/kg crizotinib.

The typical MRM chromatograms of crizotinib and IS in mouse liver and small intestine samples are shown in Figure 4. No significant interference from endogenous substances was observed at the retention times of crizotinib and the IS which were 1.31 and 1.40 min, respectively.

#### 3.2.2. Linearity and Carryover

The typical regression equations of the calibration curves were calculated by a weighted (1/*x*^2^) least-squares linear regression analysis. The calibration curves, correlation coefficients (*r*), and linear ranges of crizotinib in tissue samples are listed in Table 2. The calibration curves were linear over the concentration ranges of 20–8000 ng/mL (*r* > 0.99, *n* = 8) for crizotinib. The concentration ranges of crizotinib in this study were chosen according to the crizotinib concentration of the tissues in our pilot study. For carryover, the mass response of the blank sample at the retention time of crizotinib was less than 20% of the peak area of the lower limit of quantitation (LLOQ) sample, and no carryover effect was observed for IS.

#### 3.2.3. Accuracy and Precision

The intra- and interday precision and accuracy were calculated using one-way analysis of variance for the LLOQ and QC samples. The accuracy was demonstrated as the relative error (RE, %) of the samples, and the precision was indicated as the relative standard deviation (RSD, %). The accuracy and precision data for the determination of crizotinib in mouse tissues are shown in Table 3. The results were all within the acceptable variability limits, which demonstrated that good accuracy and precision were obtained under the current method.

#### 3.2.4. Matrix Effect and Extraction Recovery

The matrix effect was evaluated by IS-normalized matrix factors (MFs) using QC samples at the low QC (LQC) and high QC (HQC) level. The IS-normalized MF was calculated by dividing the MF of the analyte by the MF of the IS [19, 22]. The IS-normalized MF of six different batches of biological matrices at two concentration levels of crizotinib was 106.5 ± 2.2% and 101.6 ± 2.7% for the mouse liver homogenate and 102.5 ± 2.7% and 96.8 ± 3.4% for the small intestine homogenate, respectively. The results indicated that matrix effects for crizotinib and IS under the current method conditions were negligible, or the matrix effects of analytes could be compensated by the IS. The extraction recoveries of crizotinib at concentrations of 60 and 6400 ng/mL were 99.1 ± 3.0% and 88.6 ± 7.3% in the mouse liver homogenate and 75.9 ± 2.1% and 86.6 ± 3.6% for the mouse small intestine homogenate, respectively. The recovery of crizotinib in the mouse small intestine homogenate at concentration of 60 ng/mL was lower than that of the mouse liver homogenate. However, the extent of the recovery of crizotinib in the mouse small intestine sample was consistent and reproducible. Therefore, the recovery could meet the requirement of quantitative analysis for biological samples. The recoveries of IS at the concentration of 40 ng/mL in the mouse liver homogenate and the small intestine homogenate were 94.2 ± 7.2% and 92.2 ± 3.6%, respectively.

#### 3.2.5. Stability

The stability results of crizotinib are summarized in Table 4. The results revealed that crizotinib was stable in the matrices under different storage conditions. This method can be used for routine analysis.

#### 3.2.6. Dilution Integrity

The dilution integrity experiments of crizotinib were conducted at two concentration (low and high) levels. The intraprecision was lower than 10% in terms of the relative standard deviation, and the accuracy was within ± 10% in terms of the relative error for crizotinib. The intraprecision and accuracy of the dilution test were within the acceptable criteria. The results indicated that crizotinib could be assayed reliably by 5-fold diluting with the blank tissue matrix when the crizotinib concentration in samples exceeded the linear ranges of standard curves.

### 3.3. Tissue Distribution Studies

**Figure 5:**
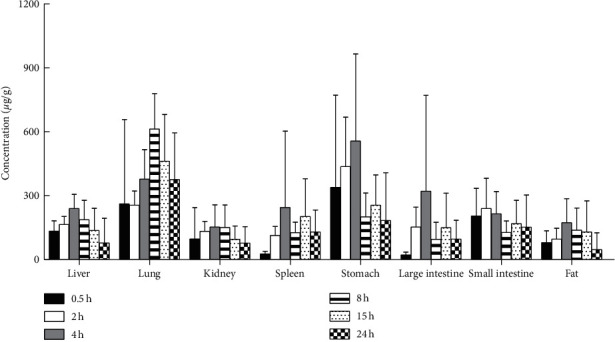
Concentrations of crizotinib in the eight tissues at 0.5 h, 2 h, 4 h, 8 h, 15 h, and 24 h after an intragastric administration of crizotinib (500 mg/kg) in healthy mice. Data are means ± SD.

**Figure 6:**
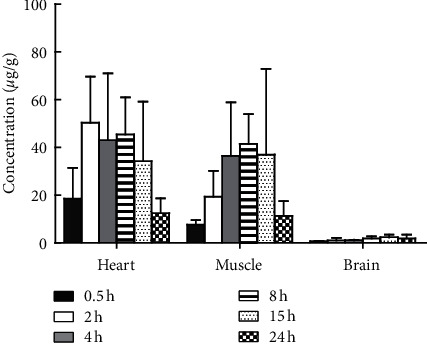
Concentrations of crizotinib in the heart, muscle, and brain at 0.5 h, 2 h, 4 h, 8 h, 15 h, and 24 h after an intragastric administration of crizotinib (500 mg/kg) in healthy mice. Data are means ± SD.

Though the FDA and European Medicines Agency (EMA) recommend that crizotinib can be taken with or without food [27], in order to minimize the impact of the gastrointestinal tract and food, the mice in this study were treated with fasted condition. After intragastric administration of 500 mg/kg crizotinib, crizotinib was widely distributed into the tested tissues. The tendency graphs of crizotinib in various mouse tissues are shown in Figures 5 and 6. The highest concentration levels of crizotinib observed in each of the tissues and the time to reach them were 50.29 ± 19.34 *μ*g/g for the heart and 239.3 ± 142.0 *μ*g/g for the small intestine at 2 h postdosing; 556.4 ± 408.6 *μ*g/g for the stomach, 239.9 ± 66.3 *μ*g/g for the liver, 153.0 ± 104.0 *μ*g/g for the kidney, 244.4 ± 357.9 *μ*g/g for the spleen, 321.2 ± 449.2 *μ*g/g for the large intestine, and 173.3 ± 112.7 *μ*g/g for the fat at 4 h postdosing; 612.4 ± 166.6 *μ*g/g for the lung and 41.39 ± 12.50 *μ*g/g for the muscle at 8 h postdosing; and 2.4 ± 1.1 *μ*g/g for the brain at 15 h postdosing. A high concentration of crizotinib was found in the gastrointestinal tract, and the results showed that the main target organs of crizotinib were the lung, liver, and spleen. A small amount of crizotinib could penetrate the blood-brain barrier. The concentrations of crizotinib in different tissues after oral administration of crizotinib (500 mg/kg) in mice have vast standard deviations, which may be caused by large differences among individuals in mice. In addition, the bimodal phenomenon of crizotinib was observed in the tissue distribution tendency graphs of some mouse tissues. The bimodal phenomenon of crizotinib might be related to the enterohepatic circulation, but we could not find the reference to support this speculation. Besides, there was no bimodal phenomenon of crizotinib in plasma according to reference [17] reported before. Therefore, further studies are required to explain this phenomenon. One limitation of the present study was that plasma quantification was not included in this LC-MS/MS method, and the results including plasma concentrations of crizotinib in mice would provide more valuable information for the clinical extrapolation.

## 4. Conclusion

In this study, a reliable LC-MS/MS method was developed and validated for the quantitative determination of crizotinib in mouse tissue samples. The method was successfully applied to a tissue distribution study of crizotinib at toxic doses in mice, which has not been reported. The results suggested that crizotinib was well distributed in the tissues detected in this study, and it was quickly eliminated in tissues, indicating that there was no long-term accumulation of crizotinib in mouse tissues. Additionally, crizotinib could cross the blood-brain barrier and was distributed in the brain tissue. The lung, liver, and spleen were the main target organs of crizotinib in the mice at the dosage of this study. This study may be useful for further research on the toxicity of crizotinib.

## Figures and Tables

**Table 1:** The differences between the current method and some previously developed methods.

Sample	Linear range (ng mL)	Retention time (min)	Column	Sample preparation method	Mobile phase	Reference
Mouse tissue	20–8000	1.31	Acquity UPLC HSS T3 column (1.8 *μ*m, 100 mm × 2.1 mm i.d.)	Protein precipitation	Methanol (solvent A) and 0.3% formic acid water solution (solvent B)	Current method
Human plasma	50–5000	4.40	Gemini C_18_ column (5.0 *μ*m, 50 × 2.0 mm i.d.)	Protein precipitation.	10 mM ammonium bicarbonate in water and 10 mM ammonium bicarbonate in methanol-water (1 : 9, v/v)	[11]
Human plasma	4–800	1.17	CORTECS® C_18_ UPLC column (dp = 1.6 *μ*m, 2.1 × 50 mm)	Solid-phase extraction	0.01% acetic acid buffer in water (solvent A) and acetonitrile added with 10% A	[12]
Human plasma	5–1000	3.80	Accucore® C_18_ column (2.1 × 50 mm, 2.6 *μ*m)	Protein precipitation	0.1% (v/v) formic acid and acetonitrile containing 0.1% (v/v) formic acid	[13]
Human plasma	0.1–1000	1.22	ACQUITY UPLC BEH C_18_ column (50 × 2.1 mm, i.d., 1.7 *μ*m)	Protein precipitation and dried under nitrogen gas	0.1% formic acid aqueous and acetonitrile/methanol (v : v, 1 : 1)	[15]
Rat plasma	1–2000	1.65	Agilent zorbax XDB C_18_ column (2.1 × 50 mm, 3.5 *μ*m)	Protein precipitation	0.1% formic acid and methanol containing 0.1% formic acid	[16]
Mouse plasma	10–10000	1.2	Acquity UPLC^®^ BEH C_18_ column (30 mm × 2.1 mm, dp = 1.7 *μ*m)	Protein precipitation	0.1% (v/v) ammonium hydroxide and methanol	[17]

**Table 2:** Standard curves of crizotinib in different biological matrices.

Biological matrices	Calibration curve	Correlation coefficient (*r*)	Linearity range (ng/mL)	LLOQ (ng/mL)
Heart	*y* = 0.00669*x* + 0.1661	0.9910	20–8000	20
Liver	*y* = 0.00588*x* + 0.09477	0.9970	20–8000	20
Spleen	*y* = 0.00686*x* + 0.1450	0.9981	20–8000	20
Lung	*y* = 0.00548*x* + 0.06471	0.9961	20–8000	20
Kidney	*y* = 0.00683*x* + 0.09242	0.9981	20–8000	20
Stomach	*y* = 0.00519*x* + 0.3896	0.9993	20–8000	20
Large intestine	*y* = 0.00487*x* + 0.08338	0.9955	20–8000	20
Small intestine	*y* = 0.00425*x* + 0.06850	0.9944	20–8000	20
Brain	*y* = 0.00729*x* + 0.1480	0.9970	20–8000	20
Muscle	*y* = 0.00648*x* + 0.2758	0.9943	20–8000	20
Fat	*y* = 0.00630*x* + 0.1874	0.9988	20–8000	20

**Table 3:** Precision and accuracy data for the quantification of crizotinib in mouse tissue homogenates (*n* = 5).

Tissues	Concentration levels (mean ± SD, ng/mL)		RSD (%)		RE (%)
	Added	Measured	Intraday	Interday	Accuracy
Liver	20	19.45 ± 2.06	10.9	8.6	−2.7
	60	62.80 ± 4.70	5.4	14.8	4.7
	600	602.2 ± 60.31	10.5	6	0.4
	6400	5875 ± 384	3.6	14.8	−8.2

Small intestine	20	19.92 ± 1.51	8.2	1.7	−0.4
	60	58.95 ± 4.16	7.3	5.8	−1.8
	600	554.6 ± 32.47	6.3	1.2	−7.6
	6400	5739 ± 256	4.7	2.2	−10.3

RSD, relative standard deviation; RE, relative error; *n*, number of replicates.

**Table 4:** Stability data of crizotinib in mouse liver and small intestines under various storage conditions (*n* = 3).

Storage conditions	Added	Liver		Small intestine	
	(ng/mL)	Measured (ng/mL)	RE (%)	Measured (ng/mL)	RE (%)
Room temperature for 8 h	60	58.38 ± 7.50	−2.7	58.71 ± 8.59	−2.1
	6400	6520.6 ± 137.9	1.9	5887.3 ± 107.9	−8.0
Autosampler for 10 h (4°C)	60	62.05 ± 3.74	3.4	64.49 ± 4.06	7.5
	6400	6844.3 ± 79.3	6.9	6682.8 ± 348.0	4.4
Three freeze-thaw cycles	60	60.10 ± 3.35	0.2	64.21 ± 0.89	7.0
	6400	6978.5 ± 291.4	9.0	6833.4 ± 92.78	6.8
Freezing for 30 days (−40°C)	60	58.97 ± 7.04	−1.7	57.70 ± 1.24	−3.8
	6400	5803.0 ± 148.3	−9.3	6758.7 ± 209.8	5.6

RSD, relative standard deviation; RE, relative error; *n*, number of replicates.

## Data Availability

The data used to support the findings of this study are included within the article.
